# Determinants of subject visit participation in a prospective cohort study of HTLV infection

**DOI:** 10.1186/1471-2288-9-19

**Published:** 2009-03-10

**Authors:** Deborah A DeVita, Mary C White, Xin Zhao, Zhanna Kaidarova, Edward L Murphy

**Affiliations:** 1 Department of Laboratory Medicine, University of California, San Francisco, California, USA; 2 Community Health Systems, School of Nursing, University of California, San Francisco, California, USA; 3 Blood Systems Research Institute, San Francisco, California, USA

## Abstract

**Background:**

Understanding participation in a prospective study is crucial to maintaining and improving retention rates. In 1990–92, following attempted blood donation at five blood centers, we enrolled 155 HTLV-I, 387 HTLV-II and 799 HTLV seronegative persons in a long-term prospective cohort.

**Methods:**

Health questionnaires and physical exams were administered at enrollment and 2-year intervals through 2004. To examine factors influencing attendance at study visits of the cohort participants we calculated odds ratios (ORs) with generalized estimated equations (GEE) to analyze fixed and time-varying predictors of study visit participation.

**Results:**

There were significant independent associations between better visit attendance and female gender (OR = 1.31), graduate education (OR = 1.86) and income > $75,000 (OR = 2.68). Participants at two centers (OR = 0.47, 0.67) and of Black race/ethnicity (OR = 0.61) were less likely to continue. Higher subject reimbursement for interview was associated with better visit attendance (OR = 1.84 for $25 vs. $10). None of the health related variables (HTLV status, perceived health status and referral to specialty diagnostic exam for potential adverse health outcomes) significantly affected participation after controlling for demographic variables.

**Conclusion:**

Increasing and maintaining participation by minority and lower socioeconomic status participants is an ongoing challenge in the study of chronic disease outcomes. Future studies should include methods to evaluate attrition and retention, in addition to primary study outcomes, including qualitative analysis of reasons for participation or withdrawal.

## Background

Understanding participation in a prospective study is crucial to maintaining and improving retention rates [1,2]. A high participant loss rate will impact the ability to draw valid conclusions. This is particularly relevant in longitudinal studies where loss of data points within or between visits can distort the relationships between measurements [3,4]. Many strong predictors of attrition, such as health problems and socioeconomic factors [5], race/ethnicity [6], and substance abuse [7] have been explored in efforts to develop strategies to maintain participation in a long term cohort study. Most studies evaluate retention by employing a survival approach, looking at the time to loss to follow-up; some have explored positive and negative predictors of retention in long-term prospective studies using mixed model statistical techniques [8]. But few have examined drop-out of study participants followed by return after missing at least one visit. Although newer statistical techniques allow analysis of data with missing data points, minimization of attrition still remains important for drawing valid research conclusions [9].

The purpose of this paper was to examine factors influencing the continued or renewed participation of subjects in a prospective longitudinal cohort study of human T-cell lymphotropic virus (HTLV) outcomes. By analyzing the large HTLV Outcomes Study (HOST) data set, we had the opportunity to investigate some uncommon but possible reasons for long-term study participation. The HTLV positive donors, with matched controls, followed in HOST since the early 1990s, permitted the examination of three research questions: first, does diagnosis with a relatively obscure virus in healthy adults impact study retention; second, does poorer health status influence participants to continue in a study, and third, does referral for specialty physician diagnostic examination, an indication of possible development of HTLV-associated disease or other adverse health outcome, contribute to long-term study enrollment. Our hypothesis was that healthy persons diagnosed with HTLV would be more likely to continue in a longitudinal study that included regular health assessments compared to HTLV negative controls. Further, we hypothesized that changes toward poorer health status regardless of HTLV status would increase the likelihood of staying in or reengaging in the study.

## Methods

### Sample

Beginning in 1990 through 1992, 155 HTLV-I seropositive, 387 HTLV-II seropositive and 799 seronegative participants were enrolled from populations of blood donors from five sites across the United States. Participants were aged 18 and older, testing either positive or negative for HTLV at the time of attempted donation. HOST data have been the source of many publications on the transmission, natural history and health outcomes of HTLV infection [10-15]. HOST is an extension of the cohort enrolled previously under the Retrovirus Epidemiology Study (REDS) and the details of HOST study design have been described elsewhere [16].

To improve the comparability of the groups, seronegative subjects were matched to HTLV seropositive subjects by age (5 year groups), sex, race/ethnicity, type of blood donation (whole blood, autologous or platelet pheresis) and blood center. A ratio of 1.5 seronegatives to HTLV seropositives was attempted, anticipating lower follow-up success with seronegative subjects. All participants were HIV seronegative. The HOST cohort included some sexual partners of HTLV seropositive donors, but they are excluded from analysis in this paper.

### Setting

HOST is a multi-center, longitudinal prospective cohort study of the health effects of infection with HTLV-I and HTLV-II occurring at five blood banks in United States cities. The five clinical and data collection sites include three American Red Cross (ARC) blood services centers: Chesapeake/Potomac (Washington/Baltimore), Southeastern Michigan (Detroit), and Southern California (Los Angeles), as well as two independent blood centers: Blood Centers of the Pacific in San Francisco, California and the Oklahoma Blood Institute in Oklahoma City, Oklahoma. Testing for HTLV was routinely done at the time of blood donation, and donors found to be seropositive were permanently deferred from blood donation prior to enrollment. Seropositive persons are not usually ill, as there is only a 1–2% risk of progressing to either of the two recognized HTLV related diseases: adult T-cell leukemia (ATL) and HTLV-associated myelopathy (HAM) [17].

### Procedures

Following enrollment and baseline data collection in 1990–92, participants have been contacted every two years to complete the three activities that comprise each visit: a health questionnaire, a basic neurologic exam, and phlebotomy for complete blood count and storage of specimens in the HOST biorepository. All activities and procedures were identical for seropostive and seronegative participants. Nurse counselors at each site were trained and monitored to perform all study activities in a standardized manner, but there was staff turnover during follow-up. The structured interview questions were asked by the study nurse and entered into a questionnaire booklet as the participant answered each question. Attempts were made to see all participants in person, but telephone interviews were accepted from participants who had moved out of state or who refused an in-person visit. An exception was the fourth study visit. Due to decreased resources, the fourth visit had a protocol modification which differed from the other visits. It consisted of an abbreviated health questionnaire completed by mail or telephone, no basic neurologic examination, and remote phlebotomy with the blood sample sent by courier to the central laboratory. The study reverted back to the original protocol when resources were restored for visits 5 and 6.

During each visit, an effort was made to limit the number of participants lost to follow-up by updating subject information for possible changes of name, address or telephone number. Participants consented to allow study personnel to search telephone directory assistance, the U.S. Postal Service forwarding service, public use databases, and credit bureau records if their previous information had changed between visits. Additionally, at each visit, participants were asked to designate a relative or friend who could be contacted to provide updated contact information or knowledge of the subject's death. In cases where the participant and designated contact person were no longer valid sources of information, a professional tracing expert was assigned to the participant with the purpose of discovering new contact information.

The health questionnaire, neurologic exam and phlebotomy were developed to screen for medical conditions or disease outcomes which might be associated with HTLV-I or HTLV-II, including ATL or HAM. Study clinicians developed an algorithm to identify abnormal responses in the health questionnaire, neurologic exam or phlebotomy results. A computer program was written to use the algorithm to screen all participant data (health questionnaire, basic neurologic exam, complete blood count and medical records) and identify/flag participants whose data were suggestive of clinical outcomes. A panel of three medical physicians with expertise in HTLV clinical and hematologic presentation met at regular intervals during each visit. The panel was blinded to participant serostatus and made decisions for participant referral to the local study physician and/or specialty physician for further diagnostic examinations in a uniform fashion.

### Data

#### Dependent Variable

The outcome of interest for this analysis is the attendance of study participants at each of the visits 2, 3, 5 and 6, following enrollment in the baseline visit 1. Data for visit 4 were excluded because of different procedures for that visit (see above). Study visit participation was defined as active if a participant completed at least a study health questionnaire either in person or by telephone, whether or not he or she completed the screening physical exam and phlebotomy.

#### Independent Variables

Our independent variables were related to health status in a natural history study. One was HTLV status (HTLV-I, HTLV-II or seronegative) measured at baseline. Perceived health status, measured by a five item Likert scale from excellent to poor, was measured at each visit. The third independent variable was referral to a specialty physician diagnostic examination, also determined at each visit. In addition to the main independent variables, another time-varying variable measured at each visit was reimbursement for interview, which changed from $10 to $25 for visits 4, 5 and 6.

#### Covariates

Fixed covariates measured at baseline were gender, age, race/ethnicity, education, annual income, ever use of injection drugs and study site. Race/ethnicity was recorded in detail (16 specific origins corresponding to risk groups for HTLV infection) but was collapsed to five for the analysis (White, Black, Hispanic, Asian and other). Educational achievement was collapsed from six categories (8^th ^grade or less; 9^th^–12^th ^grade but no diploma; high school graduate or equivalent, such as GED; some college or technical school; bachelor's degree; master's or professional degree) to four (high school or less; some college; bachelor's degree; master's or professional degree). Income was collapsed from seven categories (<$10,000; $10,000 to 19,999; $20,000 to 29,000; $30,000 to 39,999; $40,000 to 49,999; $50,000 to 74,999 and $75,000 or more) to five (< $10,000; $10,000 to 29,000; $30,000 to 49,999; $50,000 to 74,999 and > $75,000).

### Analysis

We first described the sample on baseline characteristics by HTLV status using chi-square tests comparing the percent in each category across HTLV status. In our initial analysis, we first categorized participants as taking part in visit 1 only (baseline only), in visit 1 and at least one other visit (some follow-up) or in all visits (all follow-up) by chi-square tests to compare proportions in each category.

We then used multivariate Generalized Estimating Equation (GEE) analysis to test the relationship between attendance at a study visit after baseline enrollment at visit 1 and independent variables over time. The model included the fixed (HTLV status, gender, age, race/ethnicity, education, income, ever drug use and site) and time-varying (health status at previous visit, referral to a specialty physician diagnostic examination at previous visit and reimbursement at previous visit) variables. Variables were then sequentially removed, starting with the least statistically significant. We forced two variables (HTLV status and referral for further exam) into the final model for plausibility: our hypothesis is that they were associated with participation, although they were not statistically significant in our adjusted model. Time was entered in the model as visit, and attendance at each visit was used to predict attendance at the following visit. GEE analysis does not require a balanced design (i.e., observations at all measurements for each participant), and it accommodates correlated errors due to repeated measures. We used the binomial logit function to estimate the likelihood of participation and to present the results of these tests in the form of adjusted odds ratios (OR) with 95% confidence intervals (CI). All analyses were done with SAS, version 9.0 (SAS Institute, Inc., Cary, NC).

## Results

The characteristics of the 1341 participants at baseline are shown, by HTLV status, in Table 1. HTLV-I blood donors were more likely to be Black and HTLV-II donors to be Hispanic, and both HTLV seropositive groups were observed to have lower education and lower annual income than HTLV seronegative donors.

**Table 1:** Characteristics of the HOST study sample at baseline by HTLV status, 1990–1992

Characteristic (N)	HTLV-I N (%) N = 155	HTLV-II N (%) N = 387	HTLV-Negative N (%) N = 799	Total N (%) N = 1341
Gender (1341)				
Male	44 (28)	102 (26)	257 (32)	403 (30)
Female	111 (72)	285 (74)	542 (68)	938 (70)
Age (1341)				
18–29	10 (6)	23 (6)	68 (8)	101 (8)
30–39	31 (20)	157 (41)	241 (30)	429 (32)
40–49	66 (43)	136 (35)	269 (34)	471 (35)
50–59	22 (14)	49 (13)	131 (17)	202 (15)
≥ 60	26 (17)	22 (6)	90 (11)	138 (10)
Race/Ethnicity (1327)				
White	59 (39)	138 (36)	309 (39)	506 (38)
Black	61 (40)	124 (32)	243 (31)	428 (32)
Hispanic	9 (6)	104 (27)	152 (19)	265 (20)
Asian	20 (13)	6 (2)	50 (6)	76 (6)
Other	3 (2)	10 (3)	39 (5)	52 (4)
Education (1340)				
High school or less	54 (35)	155 (40)	147 (18)	356 (27)
Some college	63 (41)	179 (46)	362 (45)	604 (45)
Bachelor's degree	25 (16)	40 (10)	179 (23)	244 (18)
Master's or professional degree	12 (8)	13 (3)	111 (14)	136 (10)
Annual income (1328)				
<$10,000	13 (8)	40 (10)	30 (4)	83 (6)
$10,000–29,999	645 (30)	130 (34)	173 (22)	348 (26)
$30,000–49,999	47 (31)	121 (31)	243 (31)	411 (31)
$50,000–74,999	29 (19)	67 (17)	204 (26)	300 (23)
≥ $75,000	18 (12)	26 (7)	142 (18)	186 (14)
Health status (1341)				
Excellent	87 (22)	313 (39)	44 (28)	444 (33)
Very good	129 (33)	329 (41)	42 (27)	500 (37)
Good	118 (30)	139 (17)	54 (35)	311 (23)
Fair & Poor	53 (14)	18 (2)	15 (10)	84 (6)
Ever used injection drugs (1338)				
No	152 (99)	294 (76)	788 (99)	1234 (92)
Yes	2 (1)	92 (24)	10 (1)	104 (8)
Site (1341)				
Chesapeake	32 (21)	51 (13)	122 (15)	205 (15)
Detroit	32 (21)	39 (10)	102 (13)	173 (13)
Los Angeles	44 (28)	206 (53)	345 (43)	595 (44)
Oklahoma City	16 (10)	23 (6)	74 (9)	113 (8)
San Francisco	31 (20)	68 (18)	156 (20)	255 (19)

Not all subjects answered every question; the number answering each question is listed with each characteristic. Percentages may not add to 100 because of rounding, and are based on those answering the question.

After recruitment and baseline data collection in visit 1, 88 (7%) participants were lost to follow-up and completed no further interviews or examinations; 51 (14%) after visit 2, 113 (31%) after visit 3, 84 (23%) after visit 5. Most of the 366 (27%) participants who dropped out were lost at the second or third visit. As some participants rejoined the study, a total of 1020 (76%) participants completed one or more follow-ups from visit 2 through visit 6, and 233 (17%) participants completed all visits. All 1341 participants were seen in person at baseline. Telephone interviews rather than in-person visits were done for 3% at visit 2, for 8% at visit 3, for all participants at visit 4 as described earlier, for 56% at visit 5, and for 40% at visit 6. Of the 1341 participants enrolled at baseline, 985 participated in visit 6 (73%). Characteristics in bivariate analysis by sociodemographic and health-related variables and study site, by these groupings of participation, are shown in Table 2.

**Table 2:** Subject participation by HTLV status and baseline characteristics

Characteristic (N)^a^	Participated in All Follow-up N (%) N = 233(17)	Participated in Some Follow-up N (%) N = 1020(76)	Participated in Baseline only^b^ N (%) N = 88(7)	Total N (100%)^c^ N = 1341	*P*
Viral status (1341)					0.07
HTLV-I	30 (19)	110 (71)	15 (10)	155	
HTLV-II	70 (18)	285 (74)	32 (8)	387	
HTLV-Negative	133 (17)	625 (78)	41 (5)	799	
Gender (1341)					0.15
Male	59 (15)	313 (78)	31 (8)	403	
Female	174 (18)	707 (75)	59 (6)	938	
Age (1341)					0.25
18–29	15 (15)	77 (76)	9 (9)	101	
30–39	74 (17)	320 (75)	35 (8)	429	
40–49	92 (20)	351 (74)	31 (6)	471	
50–59	28 (14)	165 (81)	10 (5)	202	
≥ 60	24 (17)	109 (79)	5 (4)	138	
Race/Ethnicity (1330)					0.01
White	80 (16)	402 (79)	24 (5)	506	
Black	94 (21)	298 (70)	39 (9)	428	
Hispanic	38 (14)	211 (80)	16 (6)	265	
Asian	8 (10)	62 (82)	6 (8)	76	
Other	11 (21)	37 (71)	4 (8)	52	
Education (1340)					0.05
High school or less	70 (20)	252 (71)	34 (9)	356	
Some college	103 (17)	465 (77)	37 (6)	604	
Bachelor's degree	43 (18)	192 (79)	10 (4)	244	
Master's or professional degree	17 (12)	112 (82)	7 (5)	136	
Annual income (1328)					0.03
<$10,000	11 (13)	62 (75)	10 (12)	83	
$10,000–29,999	69 (20)	248 (71)	31 (9)	348	
$30,000–49,999	68 (16)	316 (77)	27 (6)	411	
$50,000–74,999	50 (17)	236 (77)	14 (5)	300	
≥ $75,000	31 (17)	150 (81)	5 (3)	186	
Health status (1341)					0.02
Excellent	65 (15)	347 (78)	32 (7)	444	
Very good	92 (18)	383 (77)	25 (5)	500	
Good	57 (18)	235 (76)	19 (6)	311	
Fair & Poor	19 (22)	55 (64)	12 (14)	86	
Ever used IV drugs (1338)					0.21
Yes	17 (16)	76 (73)	11 (11)	104	
No	216 (18)	942 (76)	76 (6)	1234	
Site (1341)					<.001
Chesapeake	68 (33)	125 (61)	12 (6)	205	
Detroit	27 (16)	129 (75)	17 (9)	173	
Los Angeles	59 (10)	501 (84)	35 (6)	595	
Oklahoma City	27 (24)	82 (72)	4 (4)	113	
San Francisco	52 (20)	183 (72)	20 (8)	255	

^a ^Not all subjects answered every question; the number who did is listed with each characteristic. Percents may not add to 100 because of rounding, and are based on those answering the question.

^b^These subjects provided data at baseline (Visit1) only.

^c ^The total represents all who answered the question.

**Figure 1:**
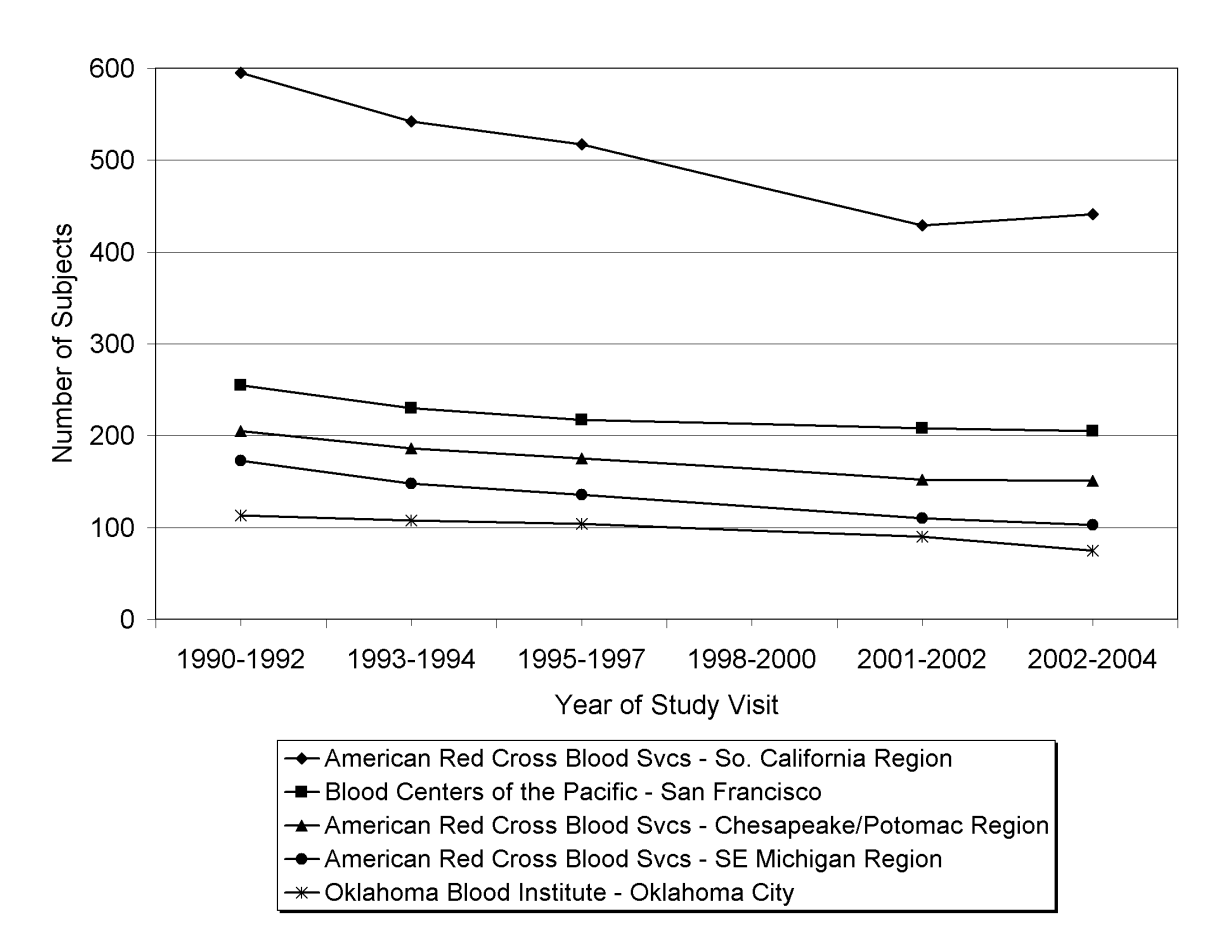
**Number of subjects submitting health questionnaires at each of 5 visits (1990–1992 through 2002–2004) by study site**.

Overall study participation by site is shown in Figure 1. Visit 4 had a telephone rather than in-person interview, and demonstrated considerably lower study participation. Further, the visit 4 health questionnaire did not include the perceived health status question. Because of the resulting loss of data and statistical power and our interest in perceived health as a predictor of participation, we examined GEE results and found no differences in effect sizes with and without visit 4 data. Table 3 and Figure1 therefore present results on study participation excluding visit 4.

**Table 3:** Predictors of subject visit participation in HOST study by bivariate and multivariate GEE analysis.

Predictor	Crude OR (95% CI)	*P*^a^	Adjusted OR (95% CI)	*P*^b^
Site				
Chesapeake	0.72 (0.48, 1.09)	0.12	0.69 (0.45, 1.04)	0.08
Detroit	0.45 (0.30, 0.67)	<0.001	0.47 (0.31, 0.71)	<0.001
Los Angeles	0.73 (0.52, 1.03)	0.08	0.67 (0.48, 0.94)	0.02
Oklahoma City	0.88 (0.56, 1.40)	0.59	0.75 (0.47, 1.19)	0.22
San Francisco	Reference		Reference	
Phase				
Visit 3 vs. Visit 2	0.63 (0.54, 0.73)	<0.001	1.34 (1.00, 1.79)	0.05
Visit 5 vs. Visit 3	0.29 (0.24, 0.35)	<0.001	0.29 (0.22, 0.39)	<0.001
Visit 6 vs. Visit 5	0.28 (0.23,0.33)	<0.001	1.38 (0.90, 2.11)	0.14
Reimbursement for interview				
$25 vs. $10	1.14 (0.95, 1.47)	0.13	1.84 (1.22, 2.77)	0.004
Gender				
Female	1.25 (0.99, 1.57)	0.05	1.31 (1.04, 1.66)	0.02
Male	Reference		Reference	
Age				
One year increase	1.00 (0.99, 1.02)	0.69	1.00 (0.99, 1.01)	0.41
Race/Ethnicity				
Black	0.51 (0.40, 0.67)	<0.001	0.61 (0.47, 0.80)	0.004
Asian	0.74 (0.44, 1.24)	0.25	0.68 (0.42, 1.10)	0.11
Hispanic	0.71 (0.52, 0.96)	0.03	0.76 (0.56, 1.04)	0.09
Other	0.59 (0.35,0.99)	0.05	0.59 (0.36, 0.99)	0.05
White	Reference		Reference	
Education				
High school or less	Reference		Reference	
Some college	1.35 (1.05, 1.73)	0.02	1.14 (0.89, 1.47)	0.29
Bachelor's degree	2.01 (1.43, 2.28)	<0.001	1.56 (1.09, 2.23)	0.02
Master's or professional degree	2.31 (1.45, 3.67)	<0.001	1.86 (1.19, 2.92)	0.01
Annual income				
<$10,000	Reference		Reference	
$10,000–29,999	1.56 (1.04, 2.34)	0.03	1.47 (0.97, 2.28)	0.07
$30,000–49,999	1.74 (1.17, 2.59)	0.01	1.57 (1.04, 2.40)	0.03
$50,000–74,999	2.52 (1.64, 3.88)	<0.001	1.96 (1.24, 3.10)	0.004
≥ $75,000	3.73 (2.25, 6.19)	<0.001	2.68 (1.58, 4.56)	0.003
Health status in previous Visit				
Excellent	1.72 (1.18,2.50)	0.01	1.20 (0.80, 1.81)	0.38
Very good	1.75 (1.22,2.52)	0.003	1.33 (0.91, 1.96)	0.14
Good	1.18 (081,1.72)	0.38	1.12 (0.85, 1.47)	0.14
Fair or Poor	Reference		Reference	
Ever used IV drugs				
Yes	1.57(1.08,2.26)	0.02	1.08 (0.83,1.42)	0.55
No	Reference		Reference	
HTLV status				
HTLV-I	0.64 (0.46, 0.89)	0.01	1.12 (0.85,1.47)	0.42
HTLV-II	0.70 (0.55, 0.88)	0.003	1.01 (0.70, 1.45)	0.96
HTLV-Negative	Reference		Reference	
Referral for further exam in previous visit				
Yes	0.86 (0.68, 1.09)	0.22	1.08 (0.83,1.42)	0.55
No	Reference		Reference	

Note: OR = odds ratio; CI= confidence interval.

^a^bivariate analysis

^b^multivariate analysis, controlling for all other values shown in the table

Among health-related variables in the bivariate analysis, HTLV-seronegatives and those with excellent or good health status were more likely to attend study visits (Table 3). However, in multiple regression analysis, neither health status nor HTLV status was associated with participation after adjusting for relevant covariates. Referral for speciality physician diagnostic exam was not a significant predictor of participation. We examined health status, which was significant in bivariate analyses but not in the multivariate model. We found that education accounted for the apparent association between health status and visit participation seen in the bivariate model.

Of the protocol-related variables, higher subject reimbursement and study site were statistically significant predictors of participation at a subsequent visit. When reimbursement was increased from $10 to $25, participants were nearly twice as likely to continue (adjusted OR 1.84). As shown by the differences in proportions, participation by study site remained significant in the GEE analysis. In particular, compared to the San Francisco site, Detroit and Los Angeles were significantly less likely to participate (adjusted OR 0.47 and 0.67, respectively).

Of the sociodemographic variables examined, there was a clear trend for socioeconomic status. Those in higher income categories were increasingly more likely to continue in the study as compared to those in the lowest income group, with those reporting $75,000 or more in annual income 2.68 times as likely to continue as those making less than $10,000. A similar trend was seen for increasing education, with those in the highest education category 1.86 times as likely as those with high school or less education to participate in study visits. Women were more likely to participate compared to men (adjusted OR 1.31), and Blacks and "other race" subjects were less likely to attend study visits as compared to Whites (adjusted OR 0.61 and 0.59, respectively).

## Discussion

The main findings of this study were that persons with higher incomes and more education were more likely to participate in study visits and men and persons of Black and other race/ethnicity were less likely to participate. Contrary to our hypothesis, HTLV seropositivity, poorer perceived health status, and referral to specialty diagnostic exam for potential adverse health outcomes did not significantly affect participation after controlling for demographic variables. Specific protocol-related characteristics did matter: study site and an increase in reimbursement were positively associated with participation.

Retention rates overall have remained high in this 12 year study of blood donors, 73% through visit 6. By virtue of selection criteria, blood donors are generally healthier than the general population. The diagnosis of a viral infection, with serious albeit rare consequences, is an unexpected consequence of blood donation. We hypothesized that being seropositive for HLTV, having poorer perceived health status, and referral for further physician examination because of possible HTLV-related disease would be associated with higher rates of overall participation and re-engagement in subsequent visits. Our data did not support these hypotheses: HTLV positive status, perceived health status, and referral for specialty physician diagnostic examination made no difference in retention or reengagement of participants. This inability to reject the null hypothesis is reassuring for the HOST study's scientific validity. Loss to follow-up related to HTLV seropositivity and the presence of adverse health outcomes, whether perceived or as a result of changes in objective health measures, could be an important source of bias in this longitudinal study.

Instead, as previously reported in the literature, demographic factors were important predictors of retention in this cohort. Males, those with lower education and lower income, and persons of color were less likely to participate in study visits. There is controversy about the effect of gender on study participation. Some studies indicate women have been shown to be more likely to consent to study participation [18] and continue in studies over time [19], others say there is no difference in participation by gender [20].

What is novel in this research is that the health status of the participants did not appear to affect visit participation. These findings are difficult to compare with other studies because of the inherent difference of these essentially healthy participants with a diagnosis as positive with a virus yet not ill, compared to participants in longitudinal studies of chronic illness. Poor health is usually predictive of dropping out of longitudinal studies [5,21,22].

Increasing and maintaining participation by underrepresented groups, who are likely to be in lower socioeconomic strata as well, is an ongoing challenge for researchers wanting to characterize health and disease for the general population [23-26]. While studies have shown that blood donors as a group have higher socioeconomic status [27], the persistent and independent influence of race/ethnicity, education and income demonstrates the continued and urgent need to develop and test strategies to encourage participation by under-represented groups.

In addition to well known sociodemographic factors, notable differences in the protocol and its implementation were important in study participation. As others have shown [28] the increase of monetary reimbursement (from $10 in visit 3 to $25 in visit 4) was positively associated with study participation. The most dramatic change in participation was seen in visit 4 when interviews were done by telephone or mail and phlebotomy was done remotely, instead of in-person interviews and phlebotomy by the study nurse. The modified approach resulted in profound decreases in participation at all centers and despite the increase in reimbursement, so for subsequent visits the study resumed in-person methods. Moreover, differences by site despite consistency in training and protocol management may have represented subtle differences in personnel and in implementation of the protocol. For example, the Los Angeles site reported that subjects moved often and required intensive tracing efforts, and that urban sprawl and the large, traffic-congested metropolitan area was cited by many subjects as a reason to drop out. Anecdotally, frequent changes in study nurses at some centers probably disrupted rapport essential to maintaining retention. These protocol and logistical observations, while consistent with common sense, remain crucial to the successful implementation of future prospective studies.

Strengths of this analysis are that the data concerned five different blood centers and a long follow-up period. HOST follows a uniform, well funded study protocol with a data coordinating center. The overall retention rate was high, allowing better measurement of differences among study groups. Limitations include the telephone follow-up in visit 4, which was addressed by excluding those data. In addition, few variables were collected specifically for the analysis of study participation. As is often the case, studies of retention are secondary analyses, peripheral to the primary research aim, and often do not have the depth or richness of data to examine the more subjective aspects of retention.

## Conclusion

In future research, investigators may wish to study various strategies to minimize participant attrition. These have been categorized by others into three areas: competence, dedication and standardized training; communication and collaborative effort between participant and researcher; and expressions of appreciation to participants [29-33]. For future longitudinal, natural history studies, researchers should consider the collection of data specifically related to study participation, including characteristics of study personnel, protocol implementation process and outcomes, changes in the study environment that could affect collection efforts, and other factors directly related to retention. Such factors may include flexible staffing hours, recommended by some to insure that the research interviews are convenient for the participant [34] and home visits, although time consuming and costly, that may have a positive impact on retention [21]. Qualitative research to better understand the range of interactions between subject and researcher may also be useful in developing testable hypotheses.

In conclusion, poor longitudinal visit participation is one of the major challenges to study validity. Our data have confirmed previous findings and suggested new insights. We recommend that future longitudinal studies incorporate specific measures of participant attrition and retention into their design, including qualitative analysis of participant-researcher interactions. In this way, real progress may be made in understanding and improving participation in studies.

## Competing interests

The authors declare that they have no competing interests.

## Authors' contributions

DAD conceived the study design and analytic methods and took overall responsibility for the paper, wrote the paper, assisted with data collection coordination, supervised data analysis and provided interpretation of the data. MCW advised on the statistical analysis and interpretation of the data, contributed to the writing and revision of the manuscript. XZ performed the statistical analysis and participated in the interpretation of findings. ZK created the analytic dataset and participated in the interpretation of the findings. ELM is the principal investigator of the cohort study HOST from which the data were derived, and contributed to all aspects of the study. All authors read and approved the final manuscript.

## Pre-publication history

The pre-publication history for this paper can be accessed here:

http://www.biomedcentral.com/1471-2288/9/19/prepub
